# P-123. Trends in Streptococcus pneumoniae Associated Meningitis, Pyogenic Arthritis, Septicemia or Pneumonia-Related Mortality in Adults Aged 25 and Above in the United States: An Analysis of Death Certificates Data Between 1999 and 2020

**DOI:** 10.1093/ofid/ofaf695.350

**Published:** 2026-01-11

**Authors:** Hamza Asif, Forest W Arnold, Saadia Ashraf

**Affiliations:** University of Louisville Hospital, Louisville, KY; University of Louisville School of Medicine, Louisville, KY; Khyber Teaching Hospital, Peshawar, Pakistan, Peshawar, North-West Frontier, Pakistan

## Abstract

**Background:**

*Streptococcus pneumoniae* is responsible for significant morbidity and mortality. Despite the availability of antibiotics and vaccination, associated mortality remains notable given incomplete vaccination, particularly among adults, and emergence of drug-resistant strains. Tracking the epidemiological trends over time of geographical variations in *S. pneumoniae* infection-related mortality in adults is relevant.

**Figure:**
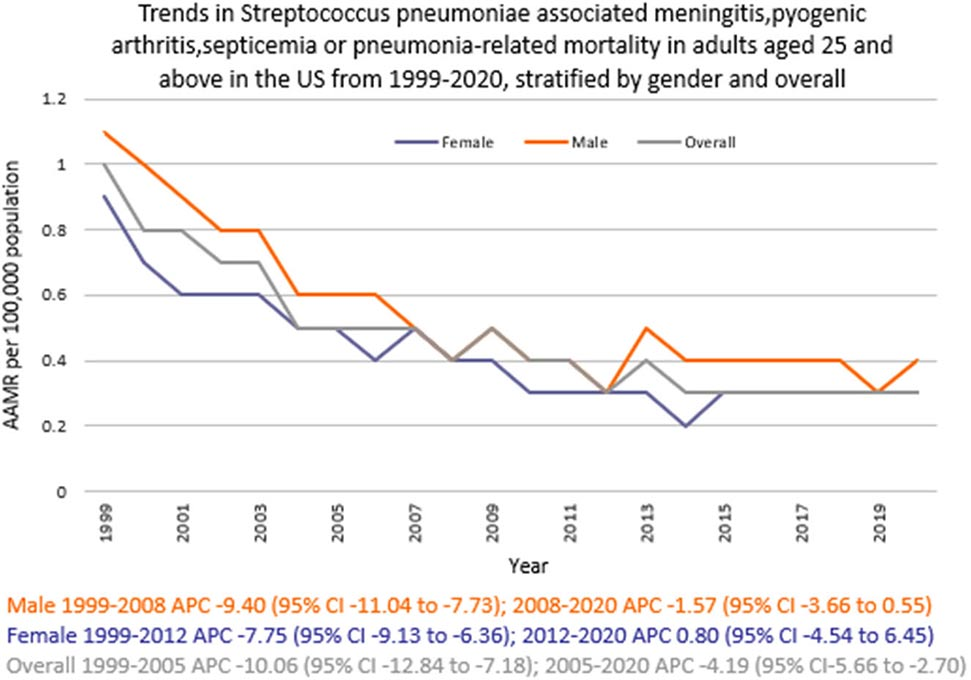

**Figure:**
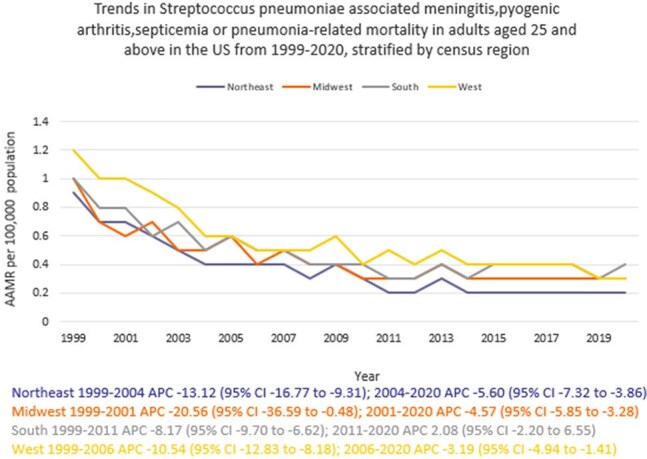

**Methods:**

Death certificate data from the CDC WONDER (Centers for Disease Control and Prevention Wide-Ranging Online Data for Epidemiologic Research) database was reviewed between 1999 and 2020 in the United States (U.S.). *S. pneumoniae*-related deaths due to meningitis, pyogenic arthritis, septicemia, or pneumonia in adults ≥ 25 years were examined using the year 2000 U.S. standard population for age standardization. Mortality rates were expressed as age-adjusted mortality rates (AAMR) per 100,000 population. Joinpoint regression was used to assess trends and calculate annual percentage change (APC), stratified by year, sex, census region, and type of geographical location of death as well as the type of facility.

**Figure:**
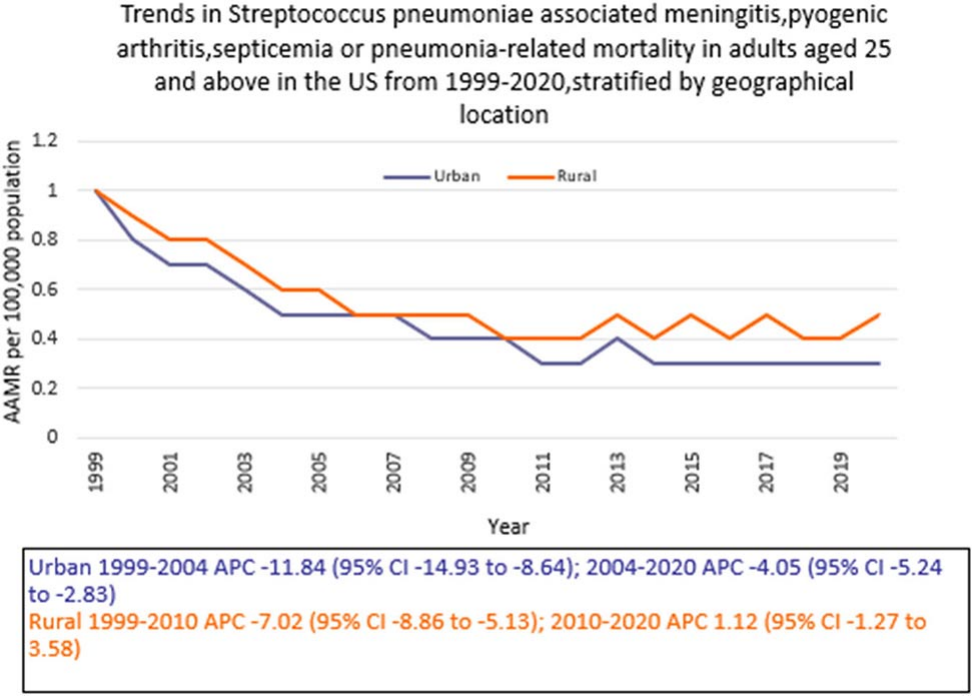

**Figure:**
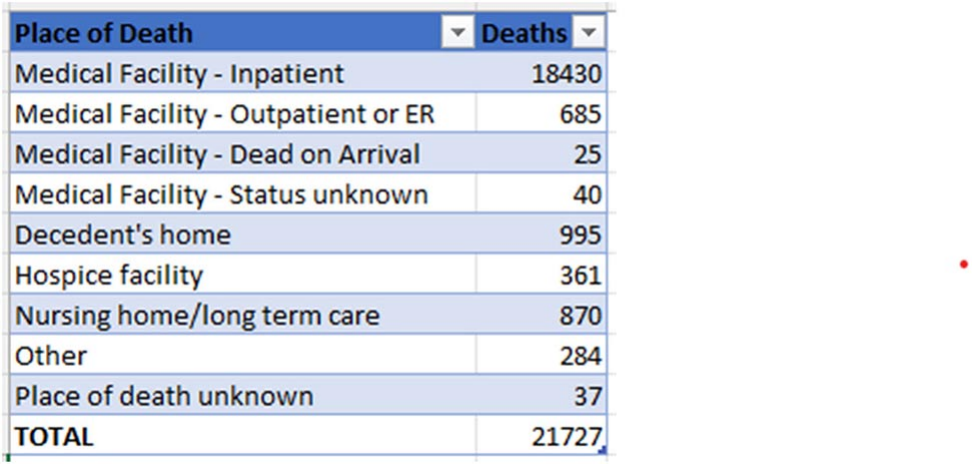

**Results:**

A total of 21,727 *S. pneumoniae*-related deaths due to select diseases occurred between 1999 and 2020. There was an abrupt decline in AAMR from 1.0 in 1999 to 0.5 in 2005 (APC -10.06; 95% CI: -12.84 to -7.18), followed by a gradual decline to 0.3 by 2020 (APC -4.19; 95% CI: -5.66 to -2.70). Men had higher AAMR overall than women (0.5 vs. 0.4). Regional variations in AAMR were also significant, with the lowest rate in the Northeast (0.3), followed by Midwest, South, and West (0.5 each). Rural areas had a higher AAMR overall (0.6) than urban (0.5). In terms of location of death, 85% died in a hospital. (Inpatient)

**Conclusion:**

From 1999 to 2020, mortality due to select *S. pneumoniae*-related infections in the U.S. declined overall, with persistent disparities particularly among men, and residents of rural areas. Addressing these disparities through targeted interventions may be crucial to reducing the burden of mortality across vulnerable groups.

**Disclosures:**

Forest W. Arnold, DO, MSc, Gilead Sciences: Grant/Research Support

